# Approaches for disease prioritization and decision-making in animal health, 2000–2021: a structured scoping review

**DOI:** 10.3389/fvets.2023.1231711

**Published:** 2023-10-06

**Authors:** Kebede Amenu, K. Marie McIntyre, Nebyou Moje, Theodore Knight-Jones, Jonathan Rushton, Delia Grace

**Affiliations:** ^1^Global Burden of Animal Diseases (GBADs) Programme, University of Liverpool, Liverpool, United Kingdom; ^2^Department of Microbiology, Immunology and Veterinary, Public Health, College of Veterinary Medicine and Agriculture, Addis Ababa University, Bishoftu, Ethiopia; ^3^Animal and Human Health Program, International Livestock Research Institute (ILRI), Addis Ababa, Ethiopia; ^4^Department of Livestock and One Health, Institute of Infection, Veterinary and Ecological Sciences, University of Liverpool, Liverpool, United Kingdom; ^5^Modelling, Evidence and Policy Group, School of Natural and Environmental Sciences, Newcastle University, Newcastle upon Tyne, United Kingdom; ^6^Department of Biomedical Sciences, College of Veterinary Medicine and Agriculture, Addis Ababa University, Bishoftu, Ethiopia; ^7^Food and Markets Department, Natural Resources Institute, University of Greenwich, London, United Kingdom; ^8^Animal and Human Health Program, International Livestock Research Institute (ILRI), Nairobi, Kenya

**Keywords:** priority setting, animal health economics, resource allocation, risk assessment, spatial analysis, animal health investment, decision-making

## Abstract

This scoping review identifies and describes the methods used to prioritize diseases for resource allocation across disease control, surveillance, and research and the methods used generally in decision-making on animal health policy. Three electronic databases (Medline/PubMed, Embase, and CAB Abstracts) were searched for articles from 2000 to 2021. Searches identified 6, 395 articles after de-duplication, with an additional 64 articles added manually. A total of 6, 460 articles were imported to online document review management software (sysrev.com) for screening. Based on inclusion and exclusion criteria, 532 articles passed the first screening, and after a second round of screening, 336 articles were recommended for full review. A total of 40 articles were removed after data extraction. Another 11 articles were added, having been obtained from cross-citations of already identified articles, providing a total of 307 articles to be considered in the scoping review. The results show that the main methods used for disease prioritization were based on economic analysis, multi-criteria evaluation, risk assessment, simple ranking, spatial risk mapping, and simulation modeling. Disease prioritization was performed to aid in decision-making related to various categories: (1) disease control, prevention, or eradication strategies, (2) general organizational strategy, (3) identification of high-risk areas or populations, (4) assessment of risk of disease introduction or occurrence, (5) disease surveillance, and (6) research priority setting. Of the articles included in data extraction, 50.5% had a national focus, 12.3% were local, 11.9% were regional, 6.5% were sub-national, and 3.9% were global. In 15.2% of the articles, the geographic focus was not specified. The scoping review revealed the lack of comprehensive, integrated, and mutually compatible approaches to disease prioritization and decision support tools for animal health. We recommend that future studies should focus on creating comprehensive and harmonized frameworks describing methods for disease prioritization and decision-making tools in animal health.

## Introduction

Livestock production is an economic process in which resources (inputs) are converted into products (outputs) (1). Livestock plays an important economic role across the world, especially in developing countries, as one of the main sources of livelihood (2). However, the livestock sector is constrained by many factors, including disease, which limits livestock production and productivity (3). Diseases negatively influence the conversion of inputs into outputs in the livestock industry, causing direct and indirect costs (4). Direct costs include mortality, reduction in the efficiency of production processes (e.g., reduced feed conversion), and reduced quantity or quality of products. Indirectly, the cost of a disease is associated with additional expenses due to disease management (vaccinate, treat, or control), public health impacts, and suboptimal use of resources, e.g., feed and water, due to infestation by disease vectors in specific localities (5).

Profitable investment in livestock production requires targeted control and prevention strategies, treatment, and surveillance of important diseases (6). Investment in mitigation of livestock disease and improvements in animal health aim to reduce production losses and minimize control costs. However, deciding where best to invest to optimize returns is complex in a livestock system that is often part of more complex systems; decisions for the farm may be made at the household level and across a range of different options at the policy level. Furthermore, there are various sociocultural and non-financial aspects to be considered in both farm- and national-level animal health control activities. A multitude of diseases and pathogens affect animals, including humans, and control efforts must be prioritized, given there are limited available resources (e.g., time, financial resources, etc.), to ensure optimal resource allocation (7, 8). The nature of the impacts of diseases varies with the pathogen and the livestock system affected. In some cases, the indirect costs of livestock diseases, especially zoonotic diseases, through their impacts on other sectors are much greater than the impact on livestock productivity. The costs of zoonoses are mainly due to non-livestock losses (9). The reaction to the presence of non-zoonotic diseases has major effects on the overall burden of disease; for example, the 2001 foot-and-mouth disease (FMD) outbreak in the UK, where there was a significant reduction in income due to shutting down the countryside and loss of tourism (10). Similarly, FMD control measures in Southern Africa had huge impacts on wildlife ecology and marginalized smallholders (11). However, endemic parasitic worm infections impact mostly through direct impacts on productivity and control costs (12), albeit the impacts of these farm-level productivity losses are rarely translated into wider economic impacts that affect downstream actors in the food system and consumers.

The basic principle of prioritizing different diseases within animal health is to maximize net benefits from allocating resources compared with the opportunity costs of alternative resource use (8). This ensures appropriate resource allocation within targeted actions, maximizing the potential benefits to animal health, public health, and the economy. The development and use of prioritization methods within human healthcare started formally several decades ago; infectious disease control and surveillance measures were targeted under the condition that not all diseases should be given equal weights for prevention and control (13). In animal health, various methods have been developed for disease priority setting and resource allocation programs. These include quantitative and qualitative approaches, such as decision tree analysis, expert opinion elicitation, risk-based assessment, semi-quantitative and quantitative scoring frameworks, and multi-criteria decision tools (8, 14–18).

However, despite its importance, the broad topic of disease prioritization for animal health priority setting lacks structure, and it exists as a range of *ad hoc* disparate tools and projects. This scoping review aims to collect the approaches used in disease prioritization and tools for animal decision-making processes. The concept of this study was developed with a view to designing a comprehensive framework for animal health decision-making and resource allocation.

## Methods

A scoping review investigating methodological approaches to animal health priority setting and decision-making tools was carried out according to the updated Preferred Reporting Items for Systematic Reviews and Meta-Analyses (PRISMA-SLR) extension for Scoping Reviews (19). In the review, prioritization was considered a process to decide the relative importance of animal health issues (e.g., the importance of diseases and their pathogenic causes, strategies for prevention, surveillance, control or research, drug treatment choices, etc.). In addition, studies that generated evidence to support decision-making, such as economic analysis, risk assessment, and spatial mapping regarding animal diseases, were also included. Therefore, the review included studies that actually or potentially assisted decisions to optimize resource use for animal disease mitigation.

### Information sources and search strategy of articles

Major bibliographic databases were systematically searched using a specific syntax for the articles retrieved. Three electronic databases (Medline/PubMed, Embase, and CAB Abstracts) were examined using the search syntax (Supplementary material 1). Keywords for the searches were identified by a detailed review of the contents of selected articles. Keywords were refined by searching for synonyms and formatted using Boolean operators. Initially, language filters were not applied, but English language literature was targeted during data extraction, and other languages were also included where articles had abstracts available in English.

### Eligibility criteria (exclusion and inclusion criteria)

Eligibility criteria defining articles to be included or excluded were developed and applied. Studies had to report “real-life” (not theoretical) application of prioritization methodologies for animal diseases, generating evidence for prioritization or decision-making by targeting the strategies identified for disease control, prevention, or surveillance at various levels (single organization or farm, local, sub-national, national, or international). Publications written in English from 1 January 2000 to 13 August 2021 (last date of search) were included. Discussion papers, reviews, and commentaries not addressing a “real-life” prioritization exercise and not implicating prioritization methods directly were not included. Studies focusing on theoretical prioritization issues (e.g., methodology development without presenting a case study on the application of the developed methods) were excluded. Prioritization studies of purely human diseases (including zoonotic diseases originating in animals but currently transmitting from human to human such as HIV/AIDS) were excluded. However, articles presenting a mix of animal and purely human diseases were considered, taking into account the animal aspects as inclusion criteria.

### Literature screening based on inclusion/exclusion criteria

Literature screening was based on the article title and abstract and was carried out by two independent reviewers, with disagreements resolved by discussion. If article abstracts were not available, the full text was screened. During the first-round screening of literature, the aforementioned eligibility criteria were used. Additionally, the geographic range covered was recorded (local, sub-national, national, and global).

In the second round of screening, retained articles were categorized based on (1) the prioritization or priority setting methods used (economic-based, simple ranking of diseases, multi-criteria, qualitative risk assessment, quantitative risk analysis, spatial (geographic) risk mapping, mix of different prioritization techniques, and other) and (2) the prioritization purpose—what the prioritization was designed to assess (disease control and prevention strategies, general importance of diseases or animal health issues, risk of disease prioritization, spatial risk mapping or analysis, disease surveillance, and research priority settings).

### Article selection process and data extraction process

The information extracted from the articles included the full bibliographic citation of the retrieved article (including authors, titles, year of publication, and journal), types of prioritizations or types of studies for evidence generation (ranging from economic, multi-criteria, spatial, risk assessment, mathematical modeling, or simple ranking), the continent of the article covered (when specified), and the geographic ranges of the focus of the articles (local to global). The different categories and sub-categories of the prioritization techniques were identified during screening and the extraction of information from the initial articles when preparing a template for full data extraction. The outputs in line with the purposes or objectives of the prioritization or evidence generation for decision-making were classified into various groups, which include general disease importance, assessment of risks (new introduction or spatial risk), and research priority setting. Species of animals and diseases or health issues mentioned in each article were also extracted. The categorization was carried out to determine how the outputs will inform users and influence decision-makers in the sense of what the prioritization process or analysis ultimately answers. The diseases, pathogens, or general health issues under consideration in each article have been extracted and presented as priorities in the form of study outputs. Species of animals considered in each article were also extracted when specified as a single species or a group of animals (e.g., ruminants, wildlife, small ruminants, equine, etc.). Species of animals described in the articles were extracted when applicable, if not presented as animals or livestock in general. The study could be focused on general livestock, specific animal species, or multiple species. Some health situations were described without mentioning diseases.

Data extraction was carried out using a form prepared in Kobotoolbox (www.kobotoolbox.org, an online data collection tool). A template for extraction was developed and pre-tested on 2–3 articles in each of the different categories. The online data capturing tool was used instead of a spreadsheet (Excel) due to its convenience for designing the formats using logics (e.g., skip patterns) and adding entries collaboratively. The online tool can accommodate complex formats, including uploading files to the system. Finally, the extracted data were exported into a spreadsheet and managed.

## Results

### Retrieved and screened articles

**Figure 1:**
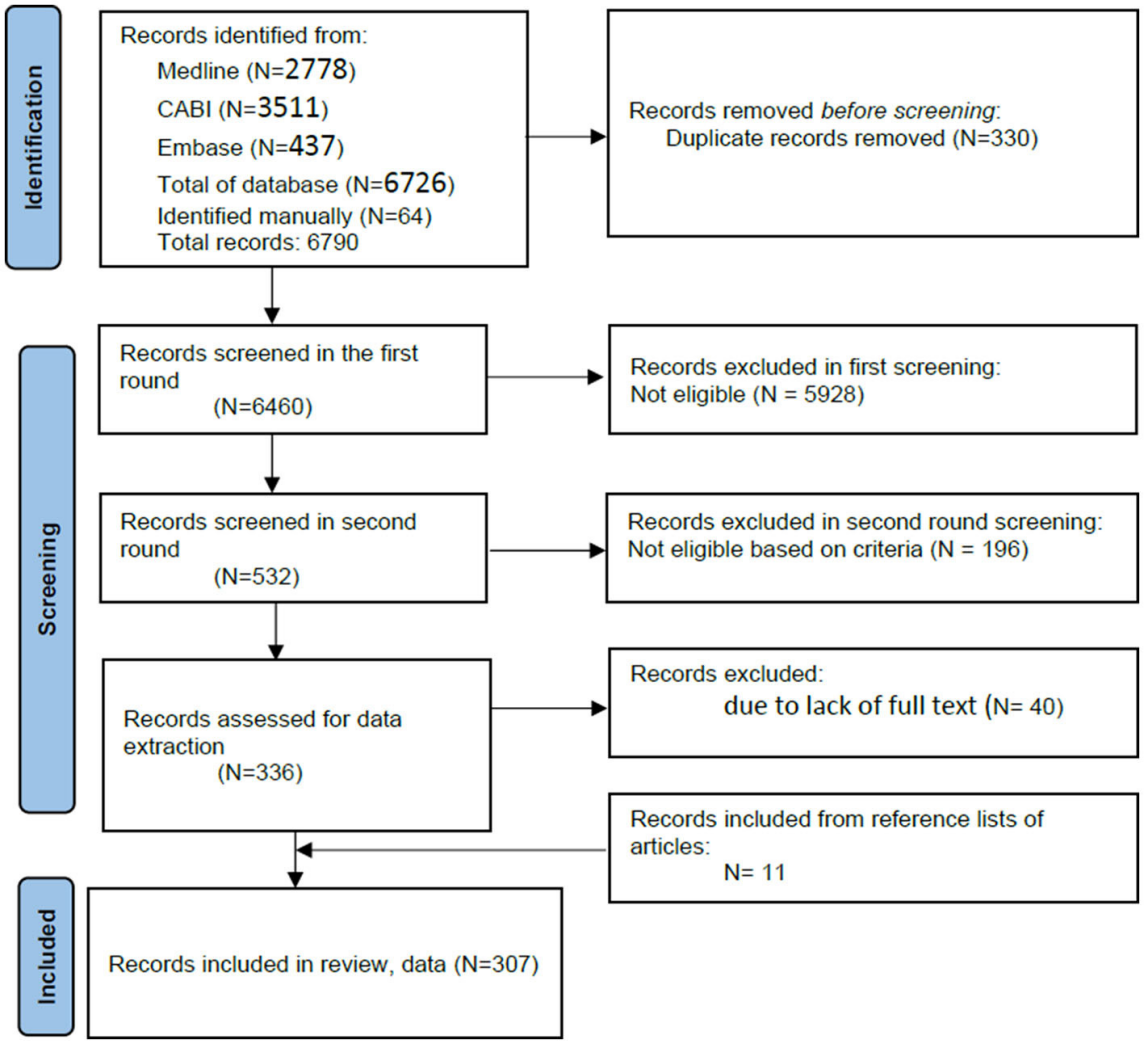
Preferred reporting items for systematic reviews and meta-analyses (PRISMA) flow diagram depicting the number of articles retrieved and screened sequentially (selected for full-text download, data extraction, and quality check) describing animal disease prioritization methods and processes (*n* = 307).

The online search identified 6, 726 articles, and upon manual searching, an additional 64 articles were added and 330 were removed due to duplicates. A total of 6, 460 articles were finally imported into online systematic review management software (20) for screening. Based on inclusion and exclusion criteria, 532 articles passed the first screening, and after a second round of screening, 336 were recommended for full review. In the preparation for data extraction, upon downloading the articles, 40 articles were excluded due to lack of full text, and 11 articles were added by snowballing from the reference lists of already identified articles. Thus, the total number of articles for final inclusion and full review was 307 (Figure 1).

### Categories of disease or animal health issues prioritization methods, yearly publication, and continental geographic distribution

Of the 307 retained articles that were fully reviewed, the results were presented under the following methods categories: (a) 96 articles on economic analysis (21–116), (b) 59 articles on multi-criteria prioritization (14, 117–173), (c) 57 articles on risk assessment (174–230), (d) 38 articles on spatial analysis (231–268), (e) 27 articles on the simple ranking of disease or animal health issues (269–295), (f) 17 articles on mathematical modeling (296–312), and (g) 13 other articles (313–325) (Table 1).

**Table 1:** Categories of disease or animal health issue prioritization methods in retained papers (*n* = 307).

**Category of prioritization methods**	**Number of articles**	**Percent**
Economic analysis	96	31.3
Multi-criteria prioritization	59	19.2
Risk assessment^*^	57	18.6
Spatial analysis	38	12.4
Simple ranking of disease or animal health issue	27	8.8
(Mathematical) modeling	17	5.5
Other^**^	13	4.2

* Risk assessment (corresponding number of articles: 29 quantitative, 21 qualitative, 7 both qualitative and quantitative); Other

** (Decision tree, H-index, and combinations of different methods).

**Figure 2:**
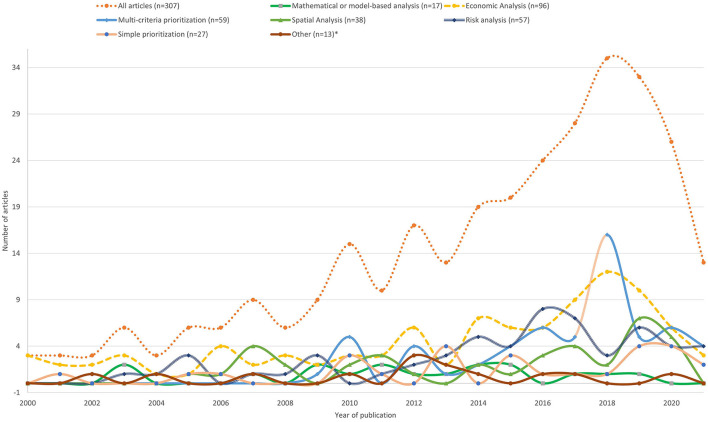
Annual (2000–2021) distribution of the articles included for data extraction disaggregated by animal health (disease) prioritization method (*n* = 307); *(decision tree, H-index, and combinations of different methods).

The annual number of publications relevant to this review increased over time, with articles using multi-criteria prioritization methods available from 2010 onward and economic analyses more evenly distributed over the duration of the two decades of publication (Figure 2).

The geographic focus of the articles was Europe (35.8%), followed by Africa (18.9%) and then North America (13.0%). European disease prioritization and evidence for decision-making studies focused on risk assessment (52.6%), and African studies mostly (63.0%) used simple ranking of diseases or health problems. Half of the articles (50.8%) focused on national-level prioritization or evidence generation for decision-making, and this figure increased to nearly 60% when multi-criteria methods were used. For articles describing spatial analysis methods, 47.5% of them focused on the national level. The number of articles describing global-level prioritizations was very limited (*n* = 13) (Table 2).

**Table 2:** Number of articles and methods used for prioritization disaggregated by continent and geographic focus.

	**Categories**	**All (*****n =*** **307)**		**MM (*****n =*** **17)**		**EA (*****n =*** **96)**		**MC (*****n =*** **59)**		**SA (*****n =*** **38)**		**RA (*****n =*** **57)**		**SR (*****n =*** **27)**		**Other (*****n =*** **13)**	
		***n***	**%**	***n***	**%**	***n***	**%**	***n***	**%**	***n***	**%**	***n***	**%**	***n***	**%**	**0**	
Geographic area	Africa	58	18.9	1	5.9	16	16.7	15	25.4	4	10.5	5	8.8	17	63.0	0	0.0
	Africa & Asia	1	0.3	0	0.0	0	0.0	0	0.0	1	2.6	0	0.0	0	0.0	0	0.0
	Africa & North America	1	0.3	0	0.0	0	0.0	1	1.7	0	0.0	0	0.0	0	0.0	0	0.0
	Asia	38	12.4	1	5.9	12	12.5	7	11.9	6	15.8	9	15.8	1	3.7	2	15.4
	Australasia	15	4.9	0	0.0	6	6.3	5	8.5	0	0.0	4	7.0	0	0.0	0	0.0
	Europe	110	35.8	7	41.2	31	32.3	16	27.1	17	44.7	30	52.6	5	18.5	4	30.8
	Global	9	2.9	0	0.0	1	1.0	0	0.0	3	7.9	0	0.0	3	11.1	2	15.4
	North America (USA and Canada)	40	13.0	2	11.8	17	17.7	10	16.9	4	10.5	6	10.5	0	0.0	1	7.7
	South America	11	3.6	1	5.9	3	3.1	1	1.7	3	7.9	1	1.8	1	3.7	1	7.7
	Not specified	24	7.8	5	29.4	10	10.4	4	6.8	0	0.0	2	3.5	0	0.0	3	23.1
Geographic range of the studies	Local	38	12.4	0	0.0	17	17.7	4	6.8	6	15.8	3	5.3	8	29.6	0	0.0
	Sub-national	20	6.5	0	0.0	5	5.2	4	6.8	5	13.2	2	3.5	3	11.1	1	7.7
	National	156	50.8	7	41.2	45	46.9	34	57.6	18	47.4	36	63.2	11	40.7	5	38.5
	Regional	34	11.1	1	5.9	6	6.3	10	16.9	3	7.9	11	19.3	2	7.4	1	7.7
	Global	13	4.2	1	5.9	2	2.1	2	3.4	4	10.5	0	0.0	3	11.1	1	7.7
	No geographic range	46	15.0	8	47.1	21	21.9	5	8.5	2	5.3	5	8.8	0	0.0	0	0.0

MM, mathematical modeling; EA, economic analysis; MC, multi-criteria prioritization; SA, spatial analysis; RA, Risk Analysis; SR, simple ranking; n, number of articles.

### Purposes of prioritization

Of the 307 articles reviewed, 39.1% were focused on prioritizations to help target disease control, prevention, or eradication strategies, and 23.0% were to aid in the identification of priority diseases to inform general organizational strategy (Table 3).

**Table 3:** The purposes of the prioritizations described in the articles presented in this scoping review (*n* = 307).

**Purposes of prioritization or analysis**	**Number of articles**	**Percent**
Disease control, prevention, or eradication strategy	120	39.1
Identification of the priority of diseases to inform general organizational strategy	72	23.0
Assessment of risk of disease introduction or occurrence	48	15.6
Identification of high-risk areas or populations (mapping)	35	11.4
Prioritization of diseases for surveillance strategies	22	7.2
Research priority setting	6	2.0

### Sub-categories of the methods of multi-criteria prioritization and economic analysis

This part of our study describes the sub-categories of prioritization and evidence-generating studies (economic analysis, multi-criteria prioritization, and risk assessment). Different sub-types of multi-criteria prioritization studies largely based on weighting techniques were identified (Table 4). The common technique applied was the Phylum applied in studies in eastern African countries. The brief definitions of the different sub-categories are described in Supplementary material 2.

**Table 4:** The types of multi-criteria prioritization methods (*n* = 59).

Multi-criteria prioritization types^*^	**Number of articles**	**Percent**
Phylum tool (OIE/WOAH)	10	16.9
Preference ranking organization method for enrichment evaluation (PROMETHEE)	5	8.5
One Health Zoonotic Disease Prioritization (OHZDP) tool	4	6.8
Analytic Hierarchy Process (AHP)	4	6.8
Weighted sum model (WSM)	4	6.8
Las Vegas technique	3	5.1
Conjoint Analysis (CA)	2	3.4
Technique for Order of Preference by Similarity to Ideal Solution (TOPSIS)	1	1.7
Multi-attribute value theory (MAVT) multi-criteria analysis	1	1.7
James Lind Alliance (JLA) Priority Setting Partnership (PSP)	1	1.7
DISCONTOOLS (DISease CONtrol TOOLS)	2	3.4
Multi-criteria Risk Ranking	2	3.4
Companion Animals multisectoriaL Interprofessional Interdisciplinary Strategic Think tank On zoonoses (CALLISTO)	1	1.7
An evidence-based decision support tool (D2R2)—Disease briefing, Decision support, Ranking and Risk assessment	1	1.7
Dairy Biosecurity Risk Evaluation Framework (D-BriEF)	1	1.7
RISKSUR evaluation (EVA) tool	1	1.7
The WHO R&D Blueprint	1	1.7
Delphi technique	4	6.8
Method not explicitly specified	11	18.6

* A brief definition of the sub-types of multi-criteria prioritization methods is presented as Supplementary material 2.

For economic analysis, cost-benefit analysis was the most commonly used economic analysis method for prioritization (48 articles), followed by cost-effectiveness analysis (34 articles). The use of partial budget analysis was reported in 12 articles, respectively (Table 5).

**Table 5:** Common economic analytical methods in animal health (*n* = 96).

**Types of economic analysis**	**Number of articles**	**Percent**
Cost-benefit analysis	48	50.0
Cost-effectiveness analysis	34	35.4
Partial budget analysis	12	12.5
Estimation of costs of disease treatments or control or economic simulation	21	21.9

Out of the total articles dealing with risk assessment, 29, 21, and 7 articles used qualitative, quantitative, and both (qualitative and quantitative), respectively.

### Prioritized diseases

The importance of different diseases was examined in prioritization exercises involving different methods (Table 6). Paratuberculosis was most commonly examined (10 out of 96 articles) within economic analyses, with rabies, brucellosis, and FMD commonly assessed in multi-criteria prioritizations. African swine fever and FMD were most often assessed in risk assessments. FMD was also the most considered disease using simple ranking methods (Table 6).

**Table 6:** The top three diseases or health (with some described by their pathogenic causes) commonly targeted using different disease prioritization methods^*^.

**Prioritization method**	**Disease/pathogen**	**Number of articles citing the disease or pathogen**
Economic analysis (*n =* 96)	Paratuberculosis	10
	Foot and mouth disease	7
	Mastitis	7
Multi-criteria analysis (*n =* 59)	Rabies	23
	Bovine brucellosis	22
	Foot and mouth disease	16
Risk assessment (*n =* 57)	African swine fever	10
	Foot and mouth disease	9
	Salmonellosis	5
Spatial mapping (analysis) (*n =* 38)	African swine fever	5
	Foot mouth disease	5
	Salmonellosis	4
Simple ranking (*n =* 27)	Foot and mouth disease	12
	Gastrointestinal parasites	7
	Anthrax	6
Mathematical (modeling) (*n =* 17)	Foot and mouth disease	3
	Paratuberculosis	2
	Highly pathogenic avian influenza	2
Other (*n =* 13)	Foot and mouth disease	2
	*Taenia solium*	2
	Echinococcosis	2
All articles	Foot and mouth disease	54
	Bovine brucellosis	40
	Rabies	39

* The full lists of diseases with methods of prioritization are presented in the Supplementary material 3.

For all articles combined, FMD (54 articles), bovine brucellosis (40 articles), and rabies (39 articles) were the common diseases considered in the articles. From the total articles, 20 assessed general animal health situations without mentioning specific diseases (Supplementary material 3).

Table 7 depicts the type of animal species or group of species considered in the articles included in the present scoping review. Accordingly, cattle are the most common animals considered by the articles, followed by pigs (Table 7).

**Table 7:** Animal species (type) considered in the articles included in the scoping review.

**Animal types (species)**	**Number of articles**	**Animal types (species)**	**Number of articles**
Cattle	92	Wild birds	2
Pigs	43	Atlantic salmon	1
Animals (livestock) (not specified)	28	Elk	1
Poultry	27	Bat	1
Small ruminants	20	Shrimp	1
Equids	12	Wild boar	1
Ruminants	10	Domesticated yaks	1
Dogs	4	Redbox	1
Wildlife (not specified)	4	Antelope	1
Camels	3	Rodent	1
Buffalo	2	Companion animal	1
Possum	2	Fish	1

## Discussion

This scoping review examined published literature describing the prioritization of animal and zoonotic diseases by reviewing a total of 307 published articles. The context of prioritization in the review was taken as studies that either explicitly prioritized the relative importance of diseases, those that prioritized animal health interventions based on one or more criteria, or those generating evidence for wider decision-making. Studies generating evidence used economic analysis, risk assessment (adverse effects of diseases assessed), spatial analysis (risk or disease distribution mapping), or modeling approaches.

In the reviewed articles, those describing economic analysis were dominant, followed by multi-criteria-based prioritizations. Disease prioritization is a complex decision-making process (8), and prioritization based on single approaches such as economic analysis and disease burden estimates may not be rigorous and inclusive enough (326). Health issues are inherently complex, involving an understanding of the economic, social, ethical, and cultural aspects of various stakeholders. These aspects need to be captured in the decision-making process. However, how these different aspects are all considered together, e.g., in multi-criteria prioritization and decision analysis, relies on arbitrary weightings of them; such analytical systems incur costs of time and understanding.

Economic analysis was widely used in prioritization studies by means of a range of approaches. Cost-benefit analysis and cost-effectiveness analysis are most commonly used in animal health, as reported in this and other studies (327). Cost-effectiveness analysis is often used for health interventions that do not have an agreed monetary value, such as human lives or animal welfare. We would expect cost-effectiveness analysis to be used more for zoonoses and diseases with high welfare implications. As an example, cost-effectiveness analysis was not used in any non-zoonotic diseases such as African swine fever, Peste des petits ruminants, and African horse sickness. However, it was used in brucellosis (11.8%), bovine tuberculosis (17.5%), and rabies (17.5%) (Supplementary material 4, raw dataset). Multi-criteria analysis was the second most common approach used for disease prioritization. Developed in the 1960s to aid complex decision-making processes within the environmental, engineering, finance, and management sciences (328, 329), multi-criteria decision analysis combines multiple, often conflicting, alternatives in order to reach a consensus on a given issue (330). The criteria for decision analysis can be measured qualitatively or quantitatively, and the technique has been relatively recently applied within animal health and, particularly, disease control (139). Multi-criteria decision analysis involves multiple steps: (1) identification of pathogens or diseases; (2) selection, weighting, and scoring of criteria; and (3) decision analysis (163). The selection and weighting of criteria according to the views of stakeholders is crucial for outputs to be appropriate (330). Within this study, the weighting methods were Preference Ranking Organization Method Enrichment of Evaluations (PROMETHEE) and Conjoint Analysis (CA) in four articles each; the specific methods used were not explicitly stated for many (34) articles.

Studies dealing with disease prioritization and those generating evidence for decision-making are increasing. This indicates the importance of such approaches in the optimization of resources for animal health and production investment. The finding that most disease prioritization studies are focused disproportionately on developed countries illustrates differences between countries in their capacities to undertake disease prioritization exercises. Several of the disease prioritization methods require expertise and resources in terms of time to complete. Available data to be used as inputs in prioritization methods can also be a reason that most studies come from developed countries. For reliable disease prioritization for resource allocation and decision-making, timely and high-quality data are required. Animal health information systems for developing countries are not well organized, constraining the practice of priority setting in animal health and the overall decision-making process.

The levels of analysis related to geographic or political administrative boundaries of the studies included in the present literature review showed that approximately half of the studies were at the national level, with minor global coverage. There are several highly infectious animal diseases that pose a global threat (transboundary diseases), potentially causing negative socioeconomic and public health consequences beyond national boundaries. This means that the burden and consequences of animal disease go beyond national boundaries; resource allocation for actions should consider such situations. In contrast, endemic diseases are often of local importance, and resources need to be allocated accordingly. This reflects the need for customizable disease prioritization and resource allocation tools that can be used in various scenarios to provide evidence-based decision-making processes for the efficient utilization of animal resources. The present scoping review showed that more complex tools (e.g., risk analysis, modeling, and economic analysis) are commonly used in more economically developed countries (e.g., Europe) compared to simple disease ranking tools being more often used in Africa. This indicates a need to build capacity for the use of complex tools in developing countries.

Various reasons behind the delivery of disease prioritization exercises were mentioned in the reviewed articles. The intention to design control, prevention, or eradication strategies by targeting single or multiple diseases was the most common reason. Overall, the ultimate intent or purpose of disease prioritization is to ensure the allocation of limited resources toward achieving the greatest benefit in improving and maintaining human and animal health (8). Apart from the relatively substantial number of articles identified in the present scoping review, the prioritization tools developed by various organizations and used for practical purposes targeting animal diseases, including zoonoses, were not commonly cited in the peer-reviewed publications identified in this review. Diseases or pathogens targeted in different studies included in the present review were quite diverse. Economic assessment was largely targeted at endemic diseases, of which, for example, *paratuberculosis* was the most common.

## Conclusion

The present scoping review covered various approaches to disease prioritization, including those supporting decision-making in animal health. Some studies focused on explicit disease prioritization, and the remaining studies generated evidence. The scoping review revealed the lack of comprehensive, integrated, and mutually compatible approaches to disease prioritization and decision support tools for animal health; this could lead to sub-optimal resource allocation. It can be concluded that there is neither a dominant tool or sets of data being used nor a comprehensive understanding of how to prioritize actions to control specific diseases. Notably, there was more variation in prioritization analysis than in economic analysis. By far, the most popular approach was cost-benefit analysis (followed by cost-effectiveness analysis) in its true meaning. There is also a complete absence of studies on what investment is needed for the livestock sector in terms of research, education, and coordination. Accordingly, this demands further work to improve disease prioritization through the integration of existing or new approaches that could be related to disease impacts as a result of generic disease risks, spatial distribution, economic impacts, and public health impacts. In the present scoping review, only published articles in English were included, and this can be a limitation of the present study. All disease prioritization outputs may not be found in published formats, and the inclusion of gray literature in possible future studies is recommended.

## Data availability statement

The original contributions presented in the study are included in the article/Supplementary material. Further inquiries can be directed to the corresponding author.

## Author contributions

KA, KM, and DG conceptualized the study. KA and KM prepared the data retrieval query protocol. TK-J, DG, and JR reviewed the data retrieval protocol. KA and NM extracted data. KA drafted the initial manuscript. All authors made critical contributions in revising the manuscript and approved the final version.
