# Supplementary material for: Approaches for disease prioritization and decision-making in animal health, 2000–2021: a structured scoping review
Source: Front Vet Sci. 2023 Oct 6;10:1231711. doi: 10.3389/fvets.2023.1231711 (PMC10593474; doi:10.3389/fvets.2023.1231711)
Supplement: Supplementary file 1 [file Table_1.docx]

Supplementary 1: Database searched for literature retrieval

| **Database** | **Search strategy/Syntax** |
| --- | --- |
| Ovid MEDLINE(R) and Epub Ahead of Print, In-Process, In-Data-Review & Other Non-Indexed Citations, Daily and Versions(R) <1946 to August 13, 2021> | 1 animal health.tw.  2 *Animal Diseases/  3 1 or 2  4 *animals, domestic/ or livestock/ or poultry/ or service animals/  5 Swine/ or livestock.tw. or Livestock/ or Cattle/  6 camels.tw. or Camelus/  7 sheep.tw. or Sheep/ or goats.tw. or Goats/  8 chicken*.tw. or Chickens/  9 crustaceans.tw. or Crustacea/ or Fishes/ or fish*.tw.  10 horses.tw. or Horses/  11 4 or 5 or 6 or 7 or 8 or 9 or 10  12 diseases.mp. or Disease/  13 (infection* or infestation* or parasit*).tw.  14 12 or 13  15 11 and 14  16 cattle diseases/ or fish diseases/ or goat diseases/ or horse diseases/ or sheep diseases/ or swine diseases/ or Poultry Diseases/ or chicken disease*.tw.  17 3 or 15 or 16  18 (control or management or research or impact or control or prevention or treatment or strategy or strategies or decision*).tw.  19 17 and 18  20 (ranking or priorities or prioritization).tw.  21 Decision Making/ or Decision Making, Organizational/  22 Health Priorities/  23 ((priorities adj2 setting) or (priority adj2 setting)).tw.  24 risk assessment.tw. or Risk Assessment/  25 (decision adj2 making).tw.  26 (Resource adj2 allocation).tw.  27 Resource Allocation/  28 (risk adj2 profiling).mp.  29 Cost-Benefit Analysis/ or Decision Trees/ or Decision tree analysis.mp.  30 Algorithms/ or weighting.mp.  31 cost effectiveness.mp. or Cost-Benefit Analysis/  32 Accountability for reasonableness.mp.  33 Health Technology Assessment.mp. or Technology Assessment, Biomedical/  34 Decision Support Techniques/ or Multi-criteria Decision Analysis.mp.  35 (Incremental cost effectiveness or Marginal Cost Abatement Curve).mp.  36 20 or 21 or 22 or 23 or 24 or 25 or 26 or 27 or 28 or 29 or 30 or 31 or 32 or 33 or 34 or 35  37 19 and 36  38 limit 37 to yr="2000 -Current" |
| Database: Embase 1947-last date, updated daily | 1 animal health.mp. or animal health/  2 *animal disease/  3 1 or 2  4 domestic animal/  5 livestock/ or cattle/  6 poultry/  7 domestic pig/  8 camels.mp. or camel/  9 sheep/ or sheep.mp.  10 goats.mp. or goat/  11 chicken/ or chickens.mp.  12 fish/ or fishes.tw.  13 horses.mp. or horse/  14 crustaceans.mp. or Crustacea/  15 4 or 5 or 6 or 7 or 8 or 9 or 10 or 11 or 12 or 13 or 14  16 diseases.mp.  17 (infection* or infestation* or parasit*).tw.  18 16 or 17  19 15 and 18  20 cattle disease/  21 fish diseases.mp. or fish disease/  22 goat diseases.mp. or goat disease/  23 sheep diseases.mp. or sheep disease/  24 horse diseases.mp. or horse disease/  25 pig diseases.mp. or swine disease/  26 poultry diseases.mp.  27 chicken diseases.mp.  28 20 or 21 or 22 or 23 or 24 or 25 or 26 or 27  29 3 or 19 or 28  30 (control or management or research or impact or control or prevention or treatment or strategy or strategies or decision*).tw.  31 29 and 30  32 (ranking or priorities or prioritization).tw.  33 decision making/  34 health priorities.mp.  35 ((priorities adj2 setting) or (priority adj2 setting)).tw.  36 risk assessment.mp. or risk assessment/  37 (decision adj2 making).tw.  38 resource allocation/  39 (Resource adj2 allocation).tw.  40 risk profiling.mp.  41 "cost benefit analysis"/  42 "decision tree"/  43 Decision tree analysis.mp.  44 algorithm/  45 weighting.mp.  46 cost effectiveness.mp. or "cost effectiveness analysis"/  47 Accountability for reasonableness.mp.  48 Health Technology Assessment.mp. or biomedical technology assessment/  49 Decision Support Techniques.mp. or decision support system/  50 multicriteria decision analysis/  51 Incremental cost effectiveness.mp.  52 Marginal Cost Abatement Curve.mp.  53 32 or 33 or 34 or 35 or 36 or 37 or 38 or 39 or 40 or 41 or 42 or 43 or 44 or 45 or 46 or 47 or 48 or 49 or 50 or 51 or 52  54 29 and 53  55 limit 54 to yr="2000 -Current" |
| CABI: CAB Abstracts | #7 Search  (#5) AND #4 and Journal Article (Document Types) and Parasites Vectors Pathogens And Biogenic Diseases Of Animals Discontinued March 2000 or Meat Producing Animals or Animal Nutrition Production Responses or Dairy Animals or Milk And Dairy Produce or Wool Producing Animals New March 2000 or Egg Producing Animals or Agricultural Economics (CABICODES) and 2021 or 2020 or 2019 or 2018 or 2017 or 2016 or 2015 or 2014 or 2013 or 2012 or 2011 or 2010 or 2009 or 2008 or 2007 or 2006 or 2005 or 2004 or 2003 or 2002 or 2001 or 2000 (Publication Years) and English (Languages) and Prion Viral Bacterial And Fungal Pathogens Of Animals New March 2000 (Exclude – CABICODES) and Non Communicable Diseases And Injuries Of Animals (Exclude – CABICODES)  #6 Search  (#5) AND #4  #5 Search  "Decision Making" or "Health Priorities" or "priority setting" or "risk assessment" (Topic) or "Resource Allocation" or "risk profiling" or "Cost-Benefit Analysis" or "Decision Tree*" or Algorithms or weighting (Topic) or "Accountability for reasonableness" or "Health Technology Assessment" or "Decision Support Techniques" (Topic)  #4 Search  ((#2) AND #1) AND TS=(control or management or research or impact or control or prevention or treatment or strategy or strategies or decision*)  #3 Search  (#2) AND #1  #2 Search  disease* or infection* or infestation* or parasit* (Topic)  #1 Search  "domestic animal*" or Swine or livestock or Cattle or camels or sheep or goats or chicken* or crustaceans or fish*or horses (Topic) |
