# Supplementary material for: Approaches for disease prioritization and decision-making in animal health, 2000–2021: a structured scoping review
Source: Front Vet Sci. 2023 Oct 6;10:1231711. doi: 10.3389/fvets.2023.1231711 (PMC10593474; doi:10.3389/fvets.2023.1231711)
Supplement: Supplementary file 2 [file Table_2.docx]

Supplementary 2: The types and description of multicriteria prioritization techniques applied in animal health priority setting and decision making

| Multi-criteria prioritization types | Description |
| --- | --- |
| Phylum tool (OIE/WOAH) | Phylum is a disease categorization and prioritization method developed by WOAH (OIE) in which diseases are categorized based on various criteria related to epidemiological profile, economic impact, human health impact and availability of control measures. The epidemiological profiles of a disease are related to types of susceptible animal species, transmission to humans or vice versa to animals, persistence of pathogens in animals/environment/wildlife, and speed and modalities of transmission. Similarly, the economic profiles are related to species affected, impact on productions herds, spatiotemporal distribution and disease effects on consumption, availability of clinical or lab diagnostics, and availability of control and prevention measures (vaccines and medical treatments). Compilations of the scores for different criteria and sub-criteria are made using Excel spreadsheet and by summing up. Overall, the prioritization tool has been used in eastern African countries to prioritize and categorize diseases. The tool was first developed in 2010 and then updated in 2015 (1). Even though not explicitly mentioned the criteria weighting technique looks like weight-sum model (WSM)- see below. |
| One Health Zoonotic Disease Prioritization (OHZDP) Tool | One Health Zoonotic Disease Prioritization (OHZDP) is a semi-quantitative tool developed in 2014 by United States CDC by considering human, animal and environmental health sectors for in prioritization process. The criteria considered related to diseases include severity of illness in humans, bioterrorism potential, economic burden of disease, capacity to collaborate, and epidemic potential (2). The weighted ranks are summed up similar to the Phylum tool. The weighting technique for OHZDP could be also MSM (see below). |
| Preference ranking organization method for enrichment evaluation (PROMETHEE) | The PROMETHEE (Preference Ranking Organization Method for Enrichment Evaluations) method is an outranking method when a given fixed number of alternatives to be ranked and selected among them based specified criteria (3). The method uses a pair-wise comparison method enabling comparison of multiple items over multiple criteria. In case of disease prioritization, limited number of diseases or pathogens are compared to each other (pairwise) based on specified criteria (4). |
| Analytic Hierarchy Process (AHP) | AHP) is also a pairwise comparison by structuring a problem for prioritization in a hierarchy of different levels constituting goal, criteria, and alternatives (5). |
| Weighted sum model (WSM) | This is a method of aggregating scores or weights. criterion weights. A simple additive result of the summation of the product of criterion scored and standardized criterion weights (which is obtained by subtracting the mean and dividing by the standard deviation of the criterion weights) is used to get final score of the alternatives to be prioritized (6) |
| Las Vegas technique | This prioritization method (Las Vegas method) is applied by asking decision makers to distribute a given total points (e.g., 100) among various criteria (7,8) |
| Conjoint Analysis (CA) | CA is used by forcing decision makers or individuals to make decisions under the conditions both desirable and undesirable characteristics of a given criteria for choice are considered. In disease prioritization, CA can be useful to determine and assess the the relative importance of key characteristics of diseases for prioritization (9) |
| Technique for Order of Preference by Similarity to Ideal Solution (TOPSIS) | TOPSIS method is used in ranking or selecting one or more alternatives) from a fixed number of alternatives with respect to multiple criteria. This is done taking into account two contrasting conditions: the positive-ideal solution (PIS) and the negative ideal solution (NIS) (10). The preferred alternative is the one with the most close to the PIS and the further to the NIS (11). |
| Multi-attribute value theory (MAVT) | MAVT is often used when the number of alternative choices available for decision-makers becoming cumbersome to complete pairwise comparison matrices (12) |
| James Lind Alliance (JLA) Priority Setting Partnership (PSP) | JLA is non-profit organized based in UK which offers a method for better aligning of health related research agendas the needs of patients, care givers and professionals through priority setting exercise following various steps |
| DISCONTOOLS ( DISease CONtrol TOOLS) | DISCONTOOLS is a disease database containing a gap analysis and prioritization model for 52 infectious diseases of animals based on six evaluation criteria (disease knowledge; impact on animal health and welfare; impact on public health; impact on wider society; impact on trade; control tools (13). |
| Multicriteria Risk Ranking | Multicriteria risk ranking is a process of prioritizing pathogens based on various criteria for further risk assessment. The process of risk ranking constitutes three main stages: initial selection of pathogens to be ranked (often by consulting national or international database or literature review), risk ranking of pathogens according to weighted criteria and qualitative assessment of pathogens against selective criteria (14). |
| [Companion Animals multisectoriaL interprofessionaL Interdisciplinary Strategic Think tank On zoonoses (CALLISTO)](https://www.sciencedirect.com/topics/agricultural-and-biological-sciences/zoonoses) | CALLISTO was developed by adopting Phylum to small animal diseases by modifying some criteria for the listing of the diseases (15). |
| An evidence-based decision support tool (‘D2R2’)- Disease briefing, Decision support, Ranking and Risk assessment | D2R2 is an evidence-based decision support tool based on 40 criteria for the scoring exotic and endemic diseases to UK towards the prioritization and management of animal diseases (16). |
| Dairy Biosecurity Risk Evaluation Framework (D-BRiEF) | D-BRiEF is a tool developed for the assessment of all risk organisms that may pose a threat to New Zealand dairy industry by following three stages which include hazard identification, multicriteria risk assessment and communication for risk management (17). |
| RISKSUR evaluation (EVA) tool | This tool was developed by the RISKSUR consortium ([www.fp7-risksur.eu](http://www.fp7-risksur.eu/)) and it is a web-based surveillance design and evaluation tool targeting the planning and implementation of integrated epidemiological and economic evaluations of surveillance systems (18). |
| The WHO R&D Blueprint | The WHO R&D Blueprint (2018) is the output of an informal expert consultation to review and update the list of priority diseases, employing a prioritization methodology which uses the Delphi technique, questionnaires, multi-criteria decision analysis, and expert review to identify relevant diseases (19) |
| Delphi technique | Delphi is a structured anonymous process to collect data and organize for decision making in areas which requires consensus among decision makers. The data collection is carried out in series of rounds with each round summarizing information presented in the previous round which is then presented again to decision makers or experts (20,21) |

1. Phylum. Listing and categorisation of priority animal diseases, including those transmissible to humans – Mission report. Colomiers: World Organisation for Animal Health (WOAH, aka OIE). (2010).

2. Rist CL, Arriola CS, Rubin C. Prioritizing zoonoses: A proposed one health tool for collaborative decision-making. *PLoS ONE* (2014) 9: doi: 10.1371/JOURNAL.PONE.0109986

3. Behzadian M, Kazemzadeh RB, Albadvi A, Aghdasi M. PROMETHEE: A comprehensive literature review on methodologies and applications. *European Journal of Operational Research* (2010) 200:198–215. doi: 10.1016/J.EJOR.2009.01.021

4. Hongoh V, Gosselin P, Michel P, Ravel A, Waaub JP, Campagna C, Samoura K. Criteria for the prioritization of public health interventions for climate-sensitive vector-borne diseases in Quebec. *PLoS ONE* (2017) 12: doi: 10.1371/journal.pone.0190049

5. Saaty TL. Decision making with the Analytic Hierarchy Process. *International Journal of Services Sciences* (2008) 1:83–98. doi: 10.1504/IJSSCI.2008.017590

6. Roberts LC, Fosgate T. Stakeholder perceptions of foot-and-mouth disease control in South Africa. (2018) 156:38–48. doi: 10.1016/j.prevetmed.2018.05.001

7. Brookes VJ, Hernández-Jover M, Neslo R, Cowled B, Holyoake P, Ward MP. Identifying and measuring stakeholder preferences for disease prioritisation: A case study of the pig industry in Australia. *Preventive Veterinary Medicine* (2014) 113:118–131. doi: 10.1016/J.PREVETMED.2013.10.016

8. Cardoen S, Van Huffel X, Berkvens D, Quoilin S, Ducoffre G, Saegerman C, Speybroeck N, Imberechts H, Herman L, Ducatelle R, et al. Evidence-based semiquantitative methodology for prioritization of foodborne zoonoses. *Foodborne pathogens and disease* (2009) 6:1083–1096. doi: 10.1089/FPD.2009.0291

9. Ng V, Sargeant JM. A Quantitative and Novel Approach to the Prioritization of Zoonotic Diseases in North America : A Public Perspective. *PLOS ONE* (2012) 7:e48519. doi: 10.1371/journal.pone.0048519

10. El Allaki F, Christensen J, Vallières A. A modified TOPSIS (Technique for Order of Preference by Similarity to Ideal Solution) applied to choosing appropriate selection methods in ongoing surveillance for Avian Influenza in Canada. *Preventive Veterinary Medicine* (2019) 165:36–43. doi: 10.1016/j.prevetmed.2019.02.006

11. Uzun B, Taiwo M, Syidanova A, Uzun Ozsahin D. The Technique For Order of Preference by Similarity to Ideal Solution (TOPSIS). (2021)25–30. doi: 10.1007/978-3-030-64765-0_4

12. Brookes VJ, Hernández-Jover M, Cowled B, Holyoake PK, Ward MP. Building a picture: Prioritisation of exotic diseases for the pig industry in Australia using multi-criteria decision analysis. *Preventive Veterinary Medicine* (2014) 113:103–117. doi: 10.1016/j.prevetmed.2013.10.014

13. O’Brien D, Scudamore J, Charlier J, Delavergne M. DISCONTOOLS : a database to identify research gaps on vaccines , pharmaceuticals and diagnostics for the control of infectious diseases of animals. *BMC Veterinary Research* (2017)1–10. doi: 10.1186/s12917-016-0931-1

14. Horigan V, De Nardi M, Simons RRL, Bertolini S, Crescio MI, Estrada-Peña A, Léger A, Maurella C, Ru G, Schuppers M, et al. Using multi-criteria risk ranking methodology to select case studies for a generic risk assessment framework for exotic disease incursion and spread through Europe. *Preventive veterinary medicine* (2018) 153:47–55. doi: 10.1016/J.PREVETMED.2018.02.013

15. Cito F, Rijks J, Rantsios AT, Cunningham AA, Baneth G, Guardabassi L, Kuiken T, Giovannini A. Prioritization of Companion Animal Transmissible Diseases for Policy Intervention in Europe. *Journal of comparative pathology* (2016) 155:S18–S26. doi: 10.1016/J.JCPA.2015.01.007

16. Gibbens JC, Frost AJ, Houston CW, Lester H, Gauntlett FA. D2R2: an evidence-based decision support tool to aid prioritisation of animal health issues for government funding. *The Veterinary record* (2016) 179: doi: 10.1136/VR.103684

17. Hodges PMD, Davidson CAMNR, Morley DPJMC. Creating a framework for the prioritization of biosecurity risks to the New Zealand dairy industry. (2018)1–11. doi: 10.1111/tbed.12848

18. Peyre M, Hoinville L, Njoroge J, Cameron A, Traon D, Goutard F, Calba C, Grosbois V, Delabouglise A, Varant V, et al. The RISKSUR EVA tool (Survtool): A tool for the integrated evaluation of animal health surveillance systems. *Preventive veterinary medicine* (2019) 173: doi: 10.1016/J.PREVETMED.2019.104777

19. Mehand MS, Al-Shorbaji F, Millett P, Murgue B. The WHO R&D Blueprint: 2018 review of emerging infectious diseases requiring urgent research and development efforts. *Antiviral research* (2018) 159:63–67. doi: 10.1016/J.ANTIVIRAL.2018.09.009

20. Stebler N, Braam P, Falzon LC. Use of a modified Delphi panel to identify and weight criteria for prioritization of zoonotic diseases in Switzerland. *Elsevier BV* (2015) doi: 10.1016/j.prevetmed.2015.05.006

21. Wentholt MTA, Cardoen S, Imberechts H, Huffel XV, Ooms BW, Frewer LJ. Defining European preparedness and research needs regarding emerging infectious animal diseases : Results from a Delphi expert consultation. (2012) 103:81–92. doi: 10.1016/j.prevetmed.2011.09.021
